# Acute paraplegia following epidural anesthesia for cesarean delivery due to asymptomatic spinal cord ependymoma hemorrhage: A case report and literature review

**DOI:** 10.1097/MD.0000000000043224

**Published:** 2025-07-11

**Authors:** Shuaibo Shi, Yuhao Liang, Axue Cheng, Junlong Wang, Chen Knien Pac-Soo, Jie Hu

**Affiliations:** a Department of Anesthesiology, Luoyang Central Hospital Affiliated to Xinxiang Medical University, Luoyang, China; b Anaesthetics, Pain Medicine and Intensive Care, Department of Surgery and Cancer, Imperial College, London, Chelsea and Westminster Hospital, London, UK; c Department of Anaesthesia, High Wycombe Hospital, Buckshealthcare NHS Trust, High Wycombe, Buckinghamshire, UK.

**Keywords:** case report, cesarean section, ependymoma, epidural anesthesia, paraplegia

## Abstract

**Rational:**

Ependymoma is the most common spinal cord tumor in the adult population, but it is, nevertheless, very rare. Most patients have no symptoms or only nonspecific symptoms. Intraspinal anesthesia is the preferred anesthetic technique for obstetric surgery because it has minimal effects on the fetus. Spinal anesthesia causing hemorrhage into spinal tumor have been reported, but very few have been for epidural anesthesia.

**Patient concerns and diagnosis:**

We describe a case of a 36-year-old female patient who presented with acute paraplegia due to after undergoing a cesarean section under epidural anesthesia. Magnetic resonance imaging suggested the possibility of subdural intratumoral hemorrhage.

**Interventions and outcomes:**

An emergency laminectomy, at T12-to-L1 level, was performed 39 hours after the cesarean section to decompress the spinal cord. An ependymoma was diagnosed after examination of the pathological specimen. She received physical rehabilitation support after surgery. The patient was left with residual motor and sensory deficits finally.

**Lessons:**

Early identification and early surgery minimize the risk of severe neurological deficit following acute spinal cord compression.

## 1. Introduction

Ependymomas are neuroepithelial tumors. They can be classified according to their anatomical location as supratentorial, infratentorial or spinal cord ependymomas. They are slightly more common in male compared with female population with an incidence of 5 per 1,000,000 and 4 per 1,000,000 respectively.^[1]^ To some extent, they are the most common spinal tumor in adults, and they account for 75% of the ependymoma in adults; in contrast, 90% of these tumors in children occur intracranially.^[2,3]^

Epidural anesthesia is used commonly for the cesarean section, although not as often as spinal anesthesia. Nevertheless, reports, in the literature, of epidural anesthesia induced acute neurological injury in patients with undiagnosed, asymptomatic spinal cord ependymomas, are rare. Herein, we describe a case occurring in a 36-year-old lady who had an epidural catheter inserted for labor analgesia and ended up having a cesarean section surgery under epidural anesthesia, due to failure to deliver vaginally. The mother developed acute paraplegia due to hemorrhage into the ependymoma. We also review the literature on previously reported cases of spinal cord ependymoma hematoma and discuss its management.

## 2. Case report

**Figure 1.**
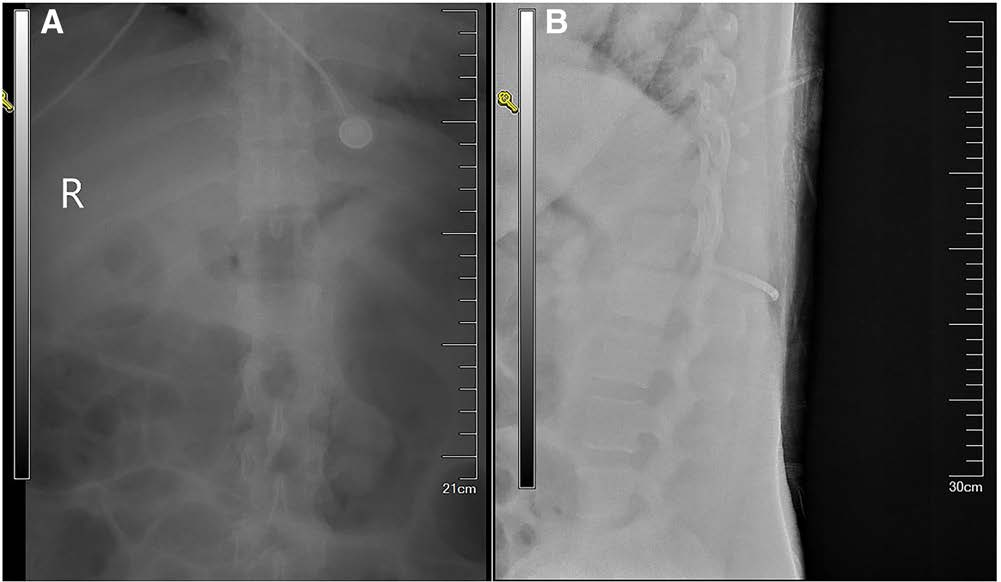
(A) Orthopantomogram of the lumbar spine; (B) lateral disk of the lumbar spine.

**Figure 2.**
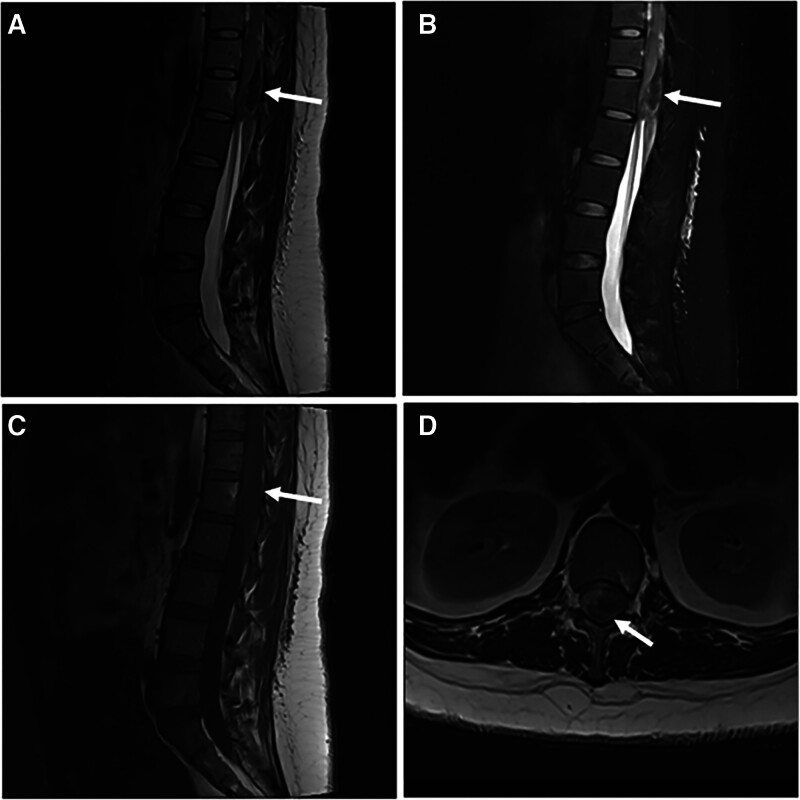
Preoperative thoracolumbar MRI scan prior to spinal cord decompression surgery. (A) Sagittal T2-weighted MRI image. (B) sagittal T2-weighted fat suppression MRI image. (C) Cagittal T1-weighted MRI image. (D) Axial T2-weighted MRI image at the level of L1. MRI revealed a heterogenous T1-isointense, T2-hyperintense lesion with mixed signals of about 8.6 × 1.6 cm at the level of T12–L2 in the subdural area, with poorly defined borders and uneven signals, and the lesion was poorly demarcated from the spinal cord cones, which were pushed out. The white arrows indicate space-occupying lesions. MRI = magnetic resonance imaging.

A 36-year-old healthy lady, who was at 38 weeks gestation, in labor ward requested a patient-controlled epidural analgesia for labor pain. She was reviewed by an anesthetist; a full history was taken, and the procedure was explained to her. She had no medical history and no family history of genetic medical disease. After ruling out contraindications related to epidural puncture, we inserted an epidural catheter using an 18G Tuohy needle, under full aseptic condition at L3 to 4 level, with the patient lying in the right lateral position. 4 cm of catheter was left in the epidural space. After excluding that the tip of the catheter was not in a vein or in the subarachnoid space, by aspiration into the catheter and administering a test dose, the patient-controlled epidural analgesia was started. 8 hours after insertion of the epidural catheter, a diagnosis of failure to progress due to fetal cephalon-pelvic disproportion, was made. A decision was taken to deliver the baby by cesarean section. The patient was transferred to the theater for the surgery. The level of the epidural block was progressively increased by administering local anesthetic, a total of 10 mL of 1% lidocaine and 5 mL of 0.75% ropivacaine, was administered via the epidural catheter. After confirmation of adequate level of block, cesarean section surgery proceeded. At the end of surgery, the epidural catheter was pulled out, the tip was inspected to confirm that the whole catheter was removed. No visible blood or mechanical damage was observed at the catheter tip. The patient was reviewed regularly by the anesthetic team and 4 hours after the last local anesthetic administration, the patient had regained complete sensory and motor functions of both lower limbs. 16 hours later, the patient complained of weakness in lifting the right lower limb upwards (muscle strength level 3). An emergency portable bedside X-ray was performed, but due to suboptimal image quality, it failed to provide diagnostic evidence of spinal canal widening or osseous destruction (Fig. 1). Consequently, an emergency magnetic resonance imaging (MRI) investigation was requested but that procedure was not available at night. The decision was made, with agreement of the family, to withhold the investigation till the following Morning. Unfortunately, 30 hours after, the patient developed complete muscle weakness of both lower limbs (muscle strength level 0) with loss of Achilles tendon reflexes. The patient was completely paraplegic on clinical examination, and she had loss bladder sensation and anal sphincter tone was weak. So, an urgent MRI was performed, and it showed a subdural space-occupying lesion at T12-to-L2 level in the spinal canal with the possibility of hemorrhage. It was difficult to tell if the lesion was a tumor or a simple hematoma. The spinal cord was displaced (Fig. 2).

**Figure 3.**
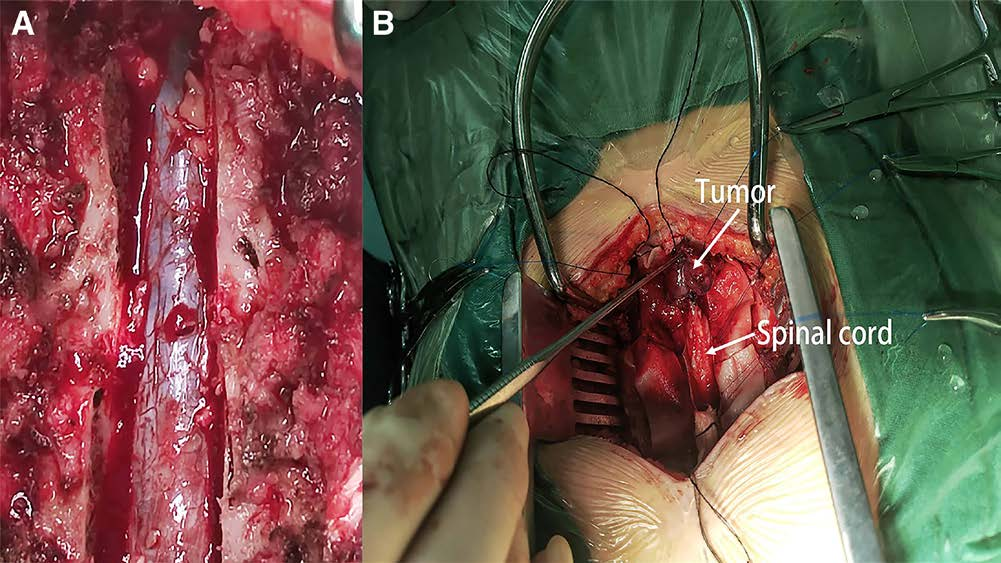
(A) Surgeon cut T12–L1 lamina to expose dura mater. (B) The dura mater was incised to reveal severe adhesion of the tumor to the spinal cord. The upper white arrow points to the tumor, and the lower 1 points to the spinal cord.

Given that the patient had developed acute paraplegia 39 hours after the cesarean section, she was taken, urgently, to theater for spinal cord decompression by a team of neurosurgeons and orthopedic surgeons. Prior to performing the surgery, attempts were made the alleviate the edema around the spinal cord by administering neurotrophic drugs (citicoline and mecobalamin), and dehydrating agents (mannitol and furosemide). Preoperative coagulation parameters were normal: PT 10.8 s (9–13 s), PTT 25.4 s (20–40 s), INR 0.83 (0.76–1.24). Elevated D-dimer (888 ng/mL, ref: 0–243 ng/mL) reflected pregnancy-related hypercoagulability, prompting perioperative enoxaparin prophylaxis. A thoracolumbar laminectomy at T12-to-L1 level was performed; after opening the dura, a 2 × 4 × 1 cm lesion was identified on the dorsum of the spinal cord. It was dark red in color, heterogenous, sticky, friable and it was adherent to the spinal cord (Fig. 3). Associated hemorrhagic clots distal to the lesion were excised alongside tumor tissue. No epidural/intramedullary hematoma was observed.

**Figure 4.**
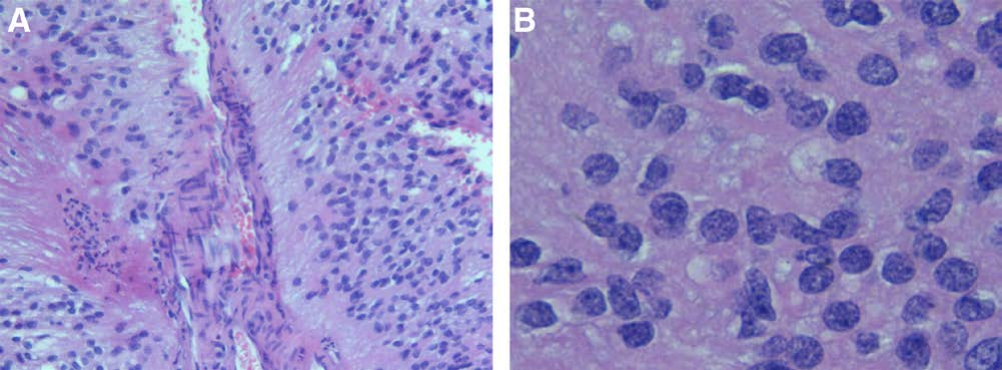
Pathological evidence shows that ependymoma conforms to WHO grade I to II. (A) Homoeopathic tumor cells are distributed in a medium to high density and arranged radially around blood vessels and fibrous protrusions form perivascular anucleate zones (pseudorosette). (B) Tumor cells form rosette. WHO = World Health Organization.

The whole tumor was resected successfully, and the specimen was sent to pathology for analysis. Histopathological examination of the specimen confirmed that it was an ependymoma with hemorrhagic changes (World Health Organization grade I and II) (Fig. 4). Immunohistochemical tests performed showed that it was epithelial membrane antigen negative, but positive for glial fibrillary acidic protein, vimentin, and S-100. The level of Ki-67 was 1%.

During her hospitalization, perioperative administration of low-molecular-weight heparin was routinely implemented for thrombosis prophylaxis. Unfortunately, lower extremity Doppler ultrasound performed on the second day after decompressive surgery revealed thrombosis in the right popliteal vein. Thrombolytic therapy was withheld due to concerns regarding bleeding risks. An inferior vena cava filter was inserted to prevent pulmonary embolism. She received physical rehabilitation support and treatment with Chinese medicine whilst in hospital. The patient showed slow recovery from the acute spinal cord injury. She regained some movements in her ankles and her toes bilaterally with a slight increase in her muscle strength, which improved from level 0 to 3, 3 months after the injury. The patient was just about able to stand with the help of crutches and she was able to void urine spontaneously; the urinary catheter was removed. She regained full sensation in her perineum but there was still residual paranesthesia in the lower limbs. Then, the patient was eventually discharged home from hospital. She continued to receive physical therapy at home and attended regular outpatient therapy.

## 3. Discussion

Spinal cord ependymomas are very rare tumors; they occur most commonly in the cervicothoracic region of the spine.^[4]^ The World Health Organization Fifth Edition Classification of central nervous system Tumors classified spinal cord ependymomas into 4 subgroups, spinal ependymoma, spinal subependymoma, myxopapillary ependymoma and spinal ependymoma with MYCN amplification respectively.^[5]^ Ependymomas, because they are slow growing tumors, do not usually cause any symptoms and if they do, they tend to be nonspecific in nature. Nevertheless, acute intratumorally hemorrhage can result in acute neurological deficits when it occurs. Common acute clinical symptoms include low-back pain, limb paralysis, superficial and deep sensory deficits in the limbs, incontinence, etc.^[2]^ Hemorrhage into an ependymoma tumor usually presents clinically as an acute neurological impairment and, unfortunately, despite prompt surgical intervention to remove the tumor completely, patients are usually left with significant neurological deficit.^[6]^ These patients often develop physical and psychological problems and live a poor quality of life afterwards. It is worth mentioning that survival rates are high for spinal cord ependymoma. Khalid et al retrospectively reported 1 year, 2 years, and 3 years survival rate after definitive diagnosis of ependymoma, that was 97.0%, 94.3%, and 93.3% respectively.^[7]^

In this case, we report a woman who developed acute paraplegia after having had an urgent cesarean section surgery performed under epidural anesthesia. We sited the epidural catheter at L3 to 4 intervertebral space and inserted 4 cm catheter into the epidural space. The subdural ependymoma hematoma occurred at T12-to-L2 level. It is unlikely that the epidural catheter caused trauma to the ependymoma resulting in hemorrhage into the tumor. Our case is among the few reported ones which describe full recovery from the epidural anesthesia with return of power and sensation into the lower limbs followed hours later by bilateral paralysis. We believe the ependymoma hematoma was caused by one of the possible scenarios: the rapid change in intra-abdominal pressure following extraction of the baby from the uterus, movements of the spine during the postoperative period, placement of the epidural catheter and injection of local anesthetics, all of these may cause changes in pressure in the spinal canal leading to hemorrhage.

This case highlights the need for vigilance and for early investigations, with an MRI scan of the spine, whenever patients complain of neurological symptoms in the lower limbs, after having had the administration of intraspinal anesthesia, to exclude an acute space-occupying lesion in the spinal cord. MRI with contrast enhancement is the preferred diagnostic method for ependymoma.^[3]^ However, due to economic and other reasons, routine preoperative MRI scan to rule out space-occupying lesions in the spinal canal remains unavailable. Furthermore, patients should be alerted to preoperative lower limb symptoms. It has been reported that a patient who suffered lower limb pain 2 months prior to the operation presented with myxopependimoma bleeding after spinal anesthesia.^[8]^ Once a diagnosis of spinal ependymoma is made, the primary treatment modality is surgical resection of the tumor,^[9]^ followed by adjuvant confocal radiation therapy to improve the cure rate.^[1]^ Unfortunately, in the case we described above, we did not offer the patient radiotherapy to the tumor after the surgery and this may influence the cancer outcome. Yet, surgical resection combined with radiotherapy does not ensure complete remission. Indeed, there is a report of recurrence of the ependymoma of up to 50% in spite of patients undergoing all of the above procedures.^[1]^ More recent treatment for the disease includes targeted therapy and immunotherapy based on the molecular biology of the ependymoma.^[1]^ It would be interesting to follow the outcome of these new treatment modalities for ependymomas. We could try adjuvant physiotherapy, in conjunction with the treatments described above, to see if that would improve the patient’s neurological outcome. Retrospectively, our management could have been optimized by performing immediate MRI upon symptom onset and initiating genetic profiling of the tumor. We did not perform the MRI immediately when the patient complained of the acute neurological symptoms. We did not do a genetic test of the ependymoma, and we did not repeat the follow-up MRI in a timely manner.

Several cases of bleeding into the ependymomas have been reported previously, following, spinal anesthesia (Table 1), and most of them were observed in obstetric anesthesia. Almost all these cases resulted in significant injury producing neurological dysfunction. However, Armstrong and colleagues reported cases of hemorrhage into the ependymomas, following spinal anesthesia with no neurological deficits.^[11]^ Consistent with what we have reported, all cases were diagnosed with intradiscal lesions using MRI scans after the appearance of symptoms. When it comes to treatment, laminectomy is recommended in all cases to remove the tumor and relieve nerve and spinal cord compression. Also in some cases, physical psychological support was administered to improve the patient’s symptoms.^[13–15]^ It is worth noting that neurological symptoms appear over a wide range of time periods, the longest period was 21 months and the shortest was immediate.^[12,13]^ Of course, this could be closely related to the amount of bleeding and the degree of spinal cord compression. As for the prognosis, the majority of patients are often left with residual lower limb muscle weakness following the acute spinal cord injury.^[10,12–15]^ Only a small proportion of patients achieve complete recovery.^[8]^

**Table 1:** Reported cases of spinal cord ependymoma hemorrhage after intraspinal anesthesia.

Study	Patient’s age and gender	Surgery	Anesthesia	Tumor (location)	Neurological signs and symptoms	Onset of manifestations	Treatment (time)	Prognosis
Jaeger et al (2002)^[10]^	28, female	Cesarean delivery	General anesthesia after 1 failed spinal anesthesia	Ependymoma (T12–L2)	Low-back pain, complete paraplegia and anesthesia from L1	12 h	T12–L2 laminectomy (8 h)	Improving motor function slowly, bowel function recovered, absent bladder function
Armstrong and Polley (2009)^[11]^	24, female	Cesarean delivery	Epidural anesthesia after 2 spinal anesthesia	Ependymoma (L2–L3)	Asymptomatic	Asymptomatic	Surgical resection of the mass (3 mo)	No surgical or neurological sequelae
Cerroni et al (2010)^[8]^	33, male	Right tibial fracture surgery	Spinal anesthesia	Myxopapillary ependymoma with a hematoma (L3–L4)	Cauda equina syndrome with urinary retention	3 d	L3–L4 laminectomy (emergency)	Completely recovered
Fournet-Fayard et al (2012)^[12]^	31, female	Eutocic vaginal delivery	Obstetric spinal anesthesia	Myxopapillary ependymoma (T1–T6)	Lower limb depletion associated with paralysis of both feet	21 mo	Surgical resection (21 mo)	Complete sequella paraplegy due to extensive spinal cord atrophy
Lee et al (2016)^[13]^	24, female	Cesarean section	Spinal anesthesia	Ependymoma (C2–T5)	Paraplegia and sensory loss	Immediately after delivery	Unilateral hemilaminectomies left C7–T1 (2 wk) and rehabilitation therapy	Residual motor deficit and cauda equina syndrome
Quintana et al (2020)^[14]^	40, female	Cesarean delivery	Spinal anesthesia	Ependymoma (T11–L5)	Low-back pain, bilateral numbness, urinary retention, difficult ambulation	6 d	T11–L5 laminectomy (13 d), physical therapy and occupational therapy	Residual motor weakness
Samargandi et al (2023)^[15]^	44, female	curettage biopsy of a cartilaginous lesion in the right proximal tibia	General anesthesia after 2 failed spinal anesthesia	Ependymoma (L2–L4)	Lumbosacral pain, bilateral lower limb weakness, hypesthesia and urinary retention	5 d	laminectomies (First 4 d, second 1 mo later), psychological support and physiotherapy	Residual deficit in muscle strength
Present report	36, female	Cesarean delivery	Epidural anesthesia	Ependymoma (T12–L2)	Paraplegia	30 h	T12–L1 laminectomy (39 h)	Residual motor and sensory dysfunction

## 4. Conclusions

Whenever a patient develops an acute, unanticipated, lower limb neurological symptom following a neuraxial anesthesia or analgesia, we must consider the possibility of an acute expanding space-occupying lesion developing in the spinal canal, including that of a spinal tumor hemorrhage as a differential diagnosis. We must send the patient for an urgent MRI of the spine to confirm the diagnosis and perform immediate laminectomy to decompress the spinal cord and nerves. Should the cause be an ependymoma tumor, the latter must be removed and followed by radiotherapy to give the patient the best chance of cure from the cancer. In conclusion, early identification and early treatment are key to better prognosis for the patient.

## Author contributions

**Conceptualization:** Jie Hu.

**Data curation:** Shuaibo Shi, Yuhao Liang.

**Investigation:** Shuaibo Shi.

**Methodology:** Jie Hu.

**Resources:** Axue Cheng.

**Supervision:** Junlong Wang.

**Writing – original draft:** Shuaibo Shi.

**Writing – review & editing:** Chen Knien Pac-Soo.
